# Pulmonary carcinoma with metastasis in a long-finned pilot whale (*Globicephala melas*)

**DOI:** 10.1186/s12917-016-0855-9

**Published:** 2016-10-11

**Authors:** Cristian M. Suárez-Santana, Carolina Fernández-Maldonado, Josué Díaz-Delgado, Manuel Arbelo, Alejandro Suárez-Bonnet, Antonio Espinosa de los Monteros, Nakita Câmara, Eva Sierra, Antonio Fernández

**Affiliations:** Division of Histology and Animal Pathology, Institute for Animal Health and Food Security, Veterinary School, University of Las Palmas de Gran Canaria, C/Transmontana, 35413 Canary Islands, Spain

**Keywords:** Pulmonary carcinoma, Pilot whale, Cetacean, Neoplasia, Tumour, Vasculogenic mimicry, Epithelial-to-mesenchymal transition

## Abstract

**Background:**

Lung cancer is the most commonly diagnosed neoplasm in humans, however this does not apply to other animal species. Living in an aquatic environment the respiratory system of cetaceans had to undergo unique adaptations in order to them to survive and cope with totally different respiratory pathogens and potentially carcinogens from those affecting humans.

**Case presentation:**

This article discusses not only macroscopical, histopathological and immunohistochemical features of a pulmonary carcinoma with disseminated metastases in a long-finned pilot whale (*Globicephala melas*), as well as the immunohistochemical analysis performed on various tissues of cetaceans belonging to the genus *Globicephala*. On the necropsy examination of the carcass, multiple pulmonary nodules and generalised thoracic lymphadenomegaly were noted. Histologically, a malignant epithelial neoplasia was identified in the lung, thoracic lymph nodes, and adrenal gland. Immunohistochemical analysis revealed a pulmonary carcinoma. Vasculogenic mimicry and epithelial-to-mesenchymal transition phenotype, as suggested by cytomorphological and immunohistochemical characteristics, were observed.

**Conclusions:**

A diagnosis of metastatic pulmonary carcinoma was determined, which to the author’s knowledge, appears to be not previously recorded in long-finned pilot whale species. This is also the first report of vasculogenic mimicry and epithelial-to-mesenchymal transition event in a spontaneous cancer from a cetacean species.

## Background

In order to survive in an aquatic environment, the respiratory system of cetaceans has undergone a complex series of morphologic adaptive changes. Some of those include pulmonary resilience and collapse during diving, presence of myoelastic sphincters, cartilaginous reinforcement of the terminal bronchi and lacking of type III brush cells, among others [1]. These adaptive capabilities may be disrupted by different pulmonary disease processes. Inflammatory conditions are one of the most prevalent disturbances affecting the lungs of free-ranging and captive cetaceans [2–4]. Other conditions, such as neoplasia, are rarely documented in this species. The only two reported cases of primary pulmonary carcinomas are one in an Amazon River dolphin (*Inia geoffrensis*) [5] and another one on a bottlenose dolphin (*Tursiops truncatus*) [6]. In humans, lung cancer is the most frequently diagnosed malignancy worldwide, encompassing mainly carcinomas (90–95 % of cases) [7]. Whilst in domestic animals, carcinoma is the most commonly reported primary pulmonary neoplasm, with two major groups: adenocarcinomas (ACA) and bronchioloalveolar carcinomas [8].

## Case presentation

This report describes gross, histopathological and immunohistochemical features of a pulmonary carcinoma with disseminated metastases in a long-finned pilot whale (LFPW) (*Globicephala melas*).

**Fig. 1:**
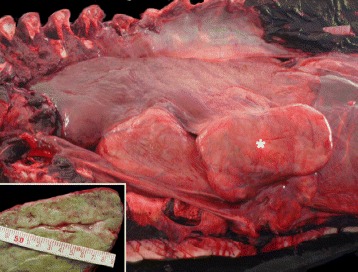
Thoracic cavity. Marked enlargement of the pulmonary lymph node (asterisk) and diffuse pulmonary atelectasis. Inset: Cut surface of the left pulmonary lymph node. Neoplastic tissue replaced the normal corticomedullary architecture of the lymph node

A 404 cm-long, adult, female LFPW stranded in Algeciras (36°05′49.5"N-5°26′33.0"W; Spain). The Stranding Network of Andalucía (Junta de Andalucía) assisted the animal but it died shortly after. A complete necropsy was performed supported by the public regional organism (Junta de Andalucía). The animal was in poor body condition. Externally, multiple, parallel cutaneous lacerations (intra/interspecific interactions) and moderate infestation by *Syncyamus* sp. were noticed. Upon dissection of the thoracic cavity, multifocal 1.6 to 4.2 cm, moderately well-defined, pale to white, firm nodules were noted throughout the lung parenchyma, while adjacent alveolar spaces were atelectatic. The mediastinal and lung-associated lymph nodes (LALN) were markedly enlarged, up to 16 × 22.5 × 12 cm (3 kg) (Fig. 1). On section, the cortex and medulla were severely replaced by a multilobulated mass of identical features to the ones found in the lung nodules. Additionally, a focal, locally extensive, lesion of 5.2 × 4.1 cm, with similar characteristic to those described in the lungs was found in the right adrenal gland, expanding the remaining non-affected glandular parenchyma. Additional gross findings included: hydropericardium, right ventricle dilatation, and severe intestinal parasitization by *Bolbosoma* sp.

For histopathological analysis, samples from skin, skeletal muscle, brain, hypophysis, thyroid gland, lungs, trachea, heart, prescapular, mediastinal and lung-associated lymph nodes, spleen, tongue, esophagus, liver, stomach, small and large intestine, pancreas, adrenal gland, uterus, ovary and mammary gland were collected and fixed in 10 % neutral buffered formalin. These samples where submitted to the Division of Histology and Animal Pathology of the Institute for Animal Health and Food Security (IUSA) in the Canary Island for processing and histopathological diagnosis. They were embedded in paraffin wax, sectioned at 5 μm and stained with haematoxylin and eosin. For immunohistochemistry, 4 μm sections of lung and LALN were obtained and immunolabeled with pancytokeratin, cytokeratins 5,7,8,18 and 20 and vimentin primary antibodies and visualized using the Dako EnVision™ system (Dako, Denmark). The immunohistochemical methodology is summarised in Table 1. Canine skin and mammary tissue were used as positive control for cytokeratin panel, whereas *Globicephala* sp. arteriolar smooth muscle was used as positive control for vimentin. Additionally, different *Globicephala* sp. tissues (*Globicephala melas* and *Globicephala macrorhynchus*) were tested for these antibodies (Table 2).

**Table 1:** Summary of immunohistochemical methodology

Antibody	Source	Host	Type	Clone	Antigen retrieval	Dilution
Pancytokeratins	Dako^a^	Mouse	Monoclonal	AE1/AE3	10 % pronase^b^	1 in 100
CK 5 + 8	Euro-Diagnostica^c^	Mouse	Monoclonal	RCK-102	10 % pronase	1 in 20
CK 8 + 18	Euro-Diagnostica	Mouse	Monoclonal	NCL-5D3	Citrate buffer^d^	1 in 20
CK7	Dako	Mouse	Monoclonal	OV-TL 12/30	Citrate buffer	1 in 50
CK 20	Dako	Mouse	Monoclonal	Ks 20.8	Citrate buffer	1 in 25
Vimentin	Dako	Mouse	Monoclonal	Vim 3B4	Citrate buffer	1 in 100

*CK* cytokeratin

^a^Dako, Glostrup, Denmark

^b^10 % pronase, 10 min at room temperature

^c^Euro-Diagnostica, Arnhem, The Netherlands

^d^Citrate buffer, pH 6.0, 20 min at 95

**Table 2:** Summary of immunohistochemical analysis of various tissues from genus Globicephala

Tissue	Specie	Cytokeratin profile
Epidermis	*G.macr*	CK5+, CK7-, CK8-, CK18-, CK20-
Bronchial/bronchiolar epithelium	*G.m and G.macr*	CK 7-, CK8-, CK18-, CK20+
Gastric epithelium	*G.macr*	CK20-
Duodenal epithelium	*G.macr*	CK20+
Arterioles (smooth muscle)	*G.macr*	Vimentin+
Pulmonary neoplasia	*G.m*	CK5+, CK7-, CK8-, CK18-, CK20+, Vimentin+

*CK* cytokeratin, *G.m Globicephala melas, G.macr Globicephala macrorhynchus*

**Fig. 2:**
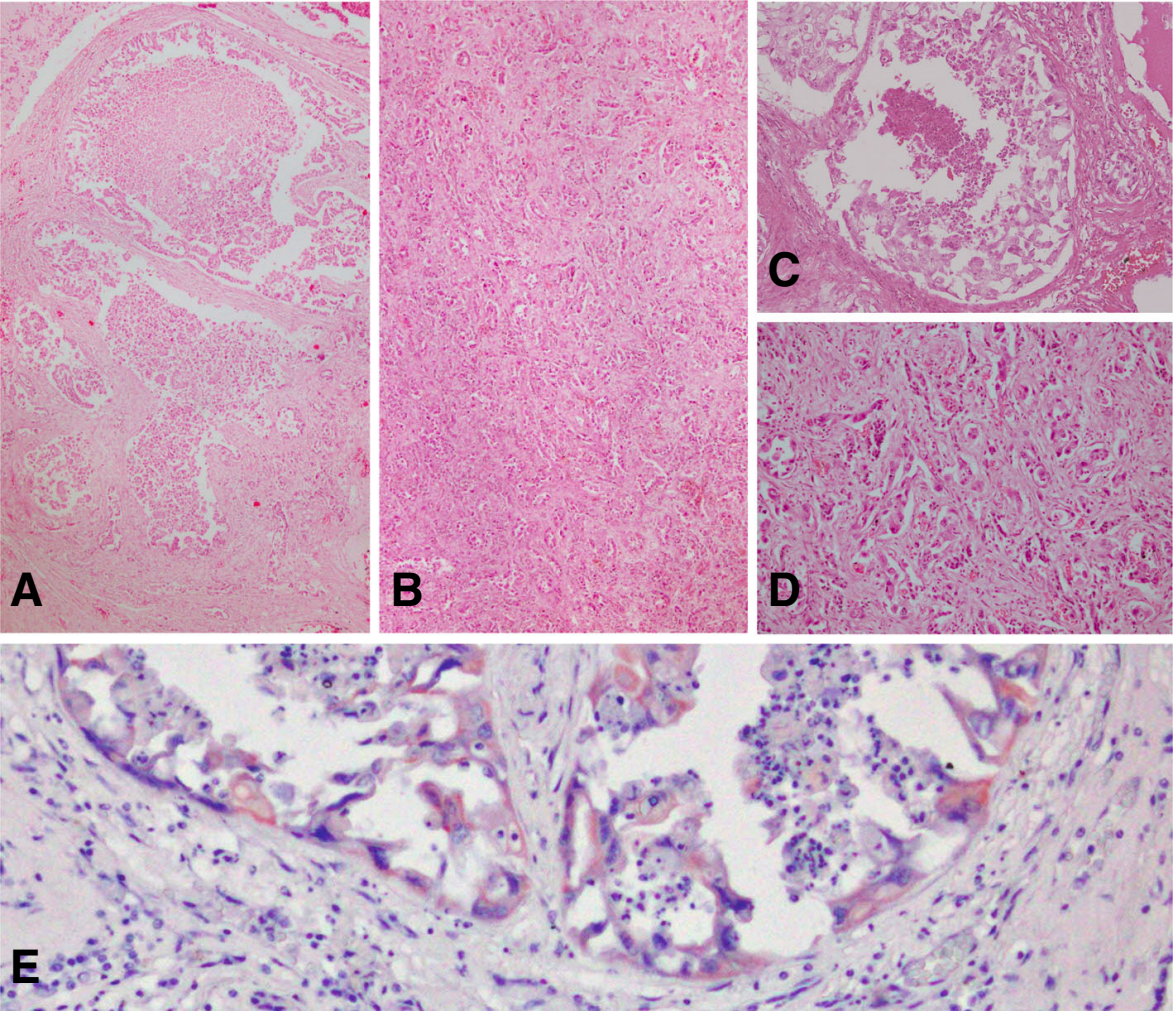
Histological and immunohistochemical characteristics of the neoplasia. Images A to D represent examples of the complex structure of the tumour: **a** bronchioloalveolar pattern (H&E, 4x); **b** adenocarcinomatous pattern (H&E, 4x). **c** Higher magnification of image A (H&E, 20x). **d** Higher magnification of image B (H&E, 20x). **e** About 90 % of neoplastic epithelial cells displayed mild, cytoplasmic and membranous labelling for cytokeratin 5 (CK 5 + 8 IHC, 40x)

**Fig. 3:**
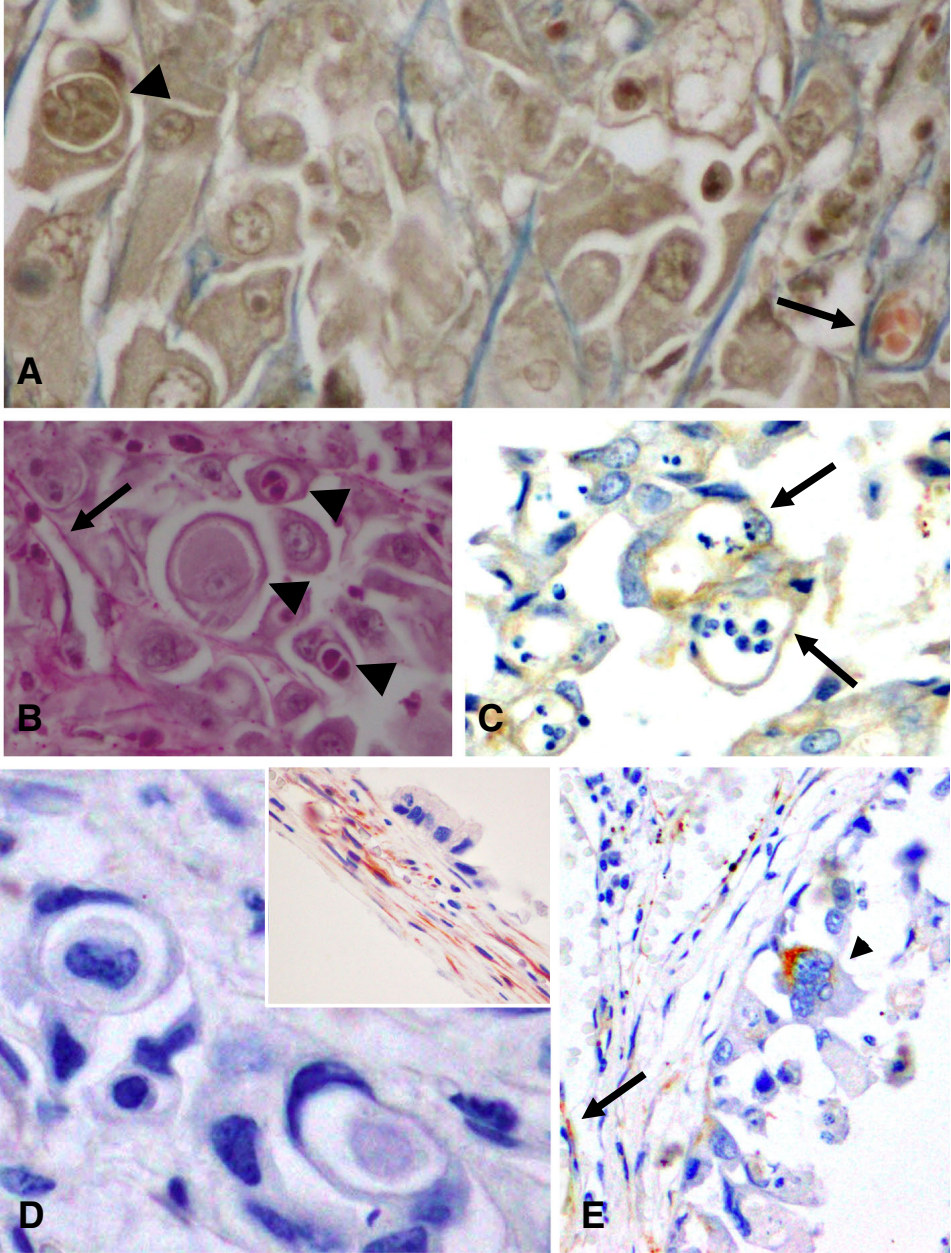
Histochemical and immunohistochemical (IHC) characteristics of vasculogenic mimicry (VM) and Epithelial-to-mesenchymal transition (EMT). **a** Masson’s Trichrome stain reveals a thin layer of stroma sustaining intratumoral capillaries (arrow), whereas VM-figures lack this support (Masson’s Trichrome, 60x). **b** The same can be visualized with the Periodic Acid-Schiff (PAS) stain, in which the basal membrane of the vessels stains PAS positive (arrow), but do not in VM-figures (PAS staining, 60x). **c** Intracytoplasmic labelling for pancytokeratin in cells forming VM, confirming their epithelial origin (AE1/AE3 IHC, 40x). **d** Intratumoral vascular endothelium consistently express vimentin (inset), whereas VM-figures do not (Vimentin IHC, 60x). **e** A multinucleated neoplastic epithelial cell displayed intracytoplasmic immunolabelling for vimentin (arrow head), feature typical of EMT. Note the staining of the vascular endothelium (arrow) functioning as internal positive control. (Vimentin IHC, 40x)

Histologically, the pulmonary parenchyma, mediastinal and LALN, and most of the right adrenal cortex were infiltrated and replaced by a multifocally coalescing, poorly demarcated, non-encapsulated, and highly infiltrative epithelial neoplasm. This displayed a complex structure with several histologic patterns encompassing adenocarcinomatous, bronchioloalveolar and adenosquamous differentiation, with areas of solid growth (Fig. 2). The tumour was characterized by epithelial cells arranged in disorganized acini, tubules and variably dilated, intercommunicating glands, resting on a thin collagenous basement membrane, and supported by thick bundles of desmoplastic (schirrous) stroma (Fig. 2). Neoplastic epithelium was monolayered ranging from flattened, cuboidal, columnar to pseudostratified (resembling bronchial epithelium) and occasionally multi-layered, with frequent papillary projections. Tumour cells had small to moderate amounts of eosinophilic, finely vacuolated cytoplasm with variably distinct borders, apical brush borders with cilia and cytoplasmic blebbing. Nuclei were irregularly round, basal to parabasal, with vesicular euchromatin and typically one prominent nucleolus. Anisocytosis and anisokaryosis were marked, and mitotic count was seven per ten 400x fields in more mitotically active areas. Karyomegaly, multinucleation, loss of polarity, vasculogenic mimicry (VM) and single cell necrosis were frequent features among tumour cells, while bizarre mitoses were scarce. Tubuloacinar and glandular lumena were filled with sloughed, degenerating and necrotic tumour cells, neutrophils, karyorhectic cellular debris, erythrocytes and proteinaceous fluid. The desmoplastic tumour stroma contained moderate numbers of lymphocytes, macrophages and few neutrophils, intermingled with areas of necrosis and haemorrhage. Vascular invasion was frequent. VM was more frequently observed in the LALN, mediastinal lymph nodes and adrenal gland metastasis, with approximately 6–10 VM-like figures per 10 high power field (40x). Histological and immunohistochemical characteristic of VM are summarized in the Fig. 3a-d.

Neoplastic cells displayed moderate intracytoplasmic and membranous immunolabeling for AE1/AE3 and CK 5 in approximately 90 % of neoplastic cells, whereas CK20 displayed weaker immunopositivity in about 60 % of the tumour cells. Vimentin immunolabeling was variable, showing intracytoplasmic, frequently yuxtanuclear, mild-to-moderate positivity in about 15 to 30 % of cancerous cells in the more labelled areas. Results of the immunohistochemical study of non-neoplastic tissues from genus *Globicephala* are depicted in Table 2. Both neoplastic cells and normal bronchial and bronchiolar epithelial cells expressed CK20, while appearing negative for CK7, CK8 and CK18 (Table 2).

Attending to gross, histological and immunohistochemical findings a primary pulmonary neoplasia with widespread metastasis was determined. Primary pulmonary epithelial neoplasia has been rarely identified in cetaceans with only two descriptions of squamous cell carcinoma (SCC) in an Amazon River dolphin (*Inia geoffrensis*) [5] and in a bottlenose dolphin (*Tursiops truncatus*) [6]. Other primary pulmonary neoplasms reported in those species include: haemangioma in bottlenose dolphin [9], common dolphin (*Delphinus delphis*) [10] and beluga whales (*Delphinapterus leucas*) [11]; fibroma in a blue whale (*Balaenoptera musculus*) and in a fin whale (*Balaenoptera physalus*) [12]; and a chondroma and lipoma in a beluga whale [13].

In veterinary medicine, adenocarcinoma is the most prevalent malignant lung tumour in dogs, cats and cattle [8]. Bronchioloalveolar carcinoma is the most prominent pattern found in sheep induced by Jaagsiekte sheep retrovirus. Whereas granular cell tumour is the most common primary lung neoplasm in horses. In humans, ACA and SCC, especially in smokers, are the most frequent lung cancers, with relatively frequent metastasis to the adrenal gland [7]. Up to 10 % of human pulmonary carcinomas display mixtures of histologic patterns (adenocarcinomatous, bronchioloalveolar and/or adenosquamous) [7], as in our case. Associated premalignant changes in humans include epithelial hyperplasia, squamous metaplasia and dysplasia which may lead to carcinoma in situ and invasive carcinoma [7]. Squamous metaplasia of the bronchial and bronchiolar epithelium has been observed in lungworm infestation in bottlenose dolphins [14] and has been speculated to be involved in neoplastic transformation in cetaceans [6]. In the present case, lungworm infestation was not grossly nor histologically apparent; however, cannot entirely be ruled out, as they may not be identifiable with chronicity or resolution [14].

Epithelial tumour cells occasionally switch from an epithelial phenotype to a mesenchymal phenotype, a phenomenon defined as epithelial-to-mesenchymal transition (EMT). In EMT, dedifferentiation with loss of epithelial characteristics and polarity occurs, frequently accompanied by vimentin expression, and acquisition of a motile mesenchymal phenotype with increased migratory behaviour and metastatic capability [15]. This phenomenon has been more widely investigated in humans than in veterinary species, and is generally associated with a poor prognosis and chemoresistence [16, 17]. Furthermore, it has not been previously reported in marine mammal neoplasia. VM is a relatively new discovered mechanism in cancer biology that consists in the formation of channels lined by neoplastic cells, adopting a pseudo-vascular disposition in order to canalize nutrients and oxygen. This contribute for tumour growth and metastasis, as cells can use these channels to colonize new locations [18]. VM can imitate blood vessels (with erythrocytes within) or more frequently lymphatic vessels (transporting white blood cells, plasma and other neoplastic cells) [18]. This feature has been noted in highly aggressive human tumours such as melanoma, inflammatory breast cancer and large cell pulmonary carcinoma [18, 19], but in animals it has only been reported in spontaneous canine mammary carcinomas [20]. In the present case, the histological and immunohistochemical characteristics of the tumour cells support VM and EMT events [15, 18], and represent the first description of these features in marine mammals’ neoplastic diseases.

## Conclusions

In conclusion, we describe a naturally occurring, highly aggressive, primary pulmonary carcinoma with adenocarcinomatous, bronchioloalveolar and adenosquamous differentiation, EMT and VM phenomena, and multiple metastases. It also represents the first primary pulmonary carcinoma described in LFPW, and contributes to expand the body of knowledge on pulmonary carcinomas biology in non-human species.

## Abbreviations

ACA: Adenocarcinoma
CK: Cytokeratin
EMT: Epithelial-to-mesenchymal transition
LALN: Lung-associated lymph node
LFPW: Long-finned pilot whale
SCC: Squamous cell carcinoma
VM: Vasculogenic mimicry

## References

[1] Piscitelli MA, Raverty SA, Lillie MA, Shadwick RE (2013). A review of cetacean lung morphology and mechanics. J Morphol.

[2] Arbelo M, De Los Monteros AE, Herraez P, Andrada M, Sierra E, Rodriguez F, Jepson P, Fernandez A (2013). Pathology and causes of death of stranded cetaceans in the Canary Islands (1999–2005). Dis Aquat Organ.

[3] Venn-Watson S, Daniels R, Smith C (2012). Thirty year retrospective evaluation of pneumonia in a bottlenose dolphin Tursiops truncatus population. Dis Aquat Organ.

[4] Jepson PD, Baker JR, Kuiken T, Simpson VR, Kennedy S, Bennett PM (2000). Pulmonary pathology of harbour porpoises (Phocoena phocoena) stranded in England and Wales between 1990 and 1996. Vet Rec.

[5] Geraci JR, Palmer JP, Aubin DJ (1897). Tumors in cetaceans: analysis and new findings. Can J Fish Aquat Sci.

[6] Ewing RY, Mignucci-Giannoni AA (2003). A poorly differentiated pulmonary squamous cell carcinoma in a free-ranging Atlantic bottlenose dolphin (Tursiops truncatus). J Vet Diagn Invest.

[7] Husain AN, Kumar V, Abbas AK, Aster JC (2015). The lung. Robbins and cotran pathologic basis of disease.

[8] Caswell LJ, Williams KJ, Grant MM (2007). Respiratory system. Jubb, kennedy & palmer’s pathology of domestic animals.

[9] Turnbull BS, Cowan DF (1999). Angiomatosis, a newly recognized disease in Atlantic bottlenose dolphins (*Tursiops truncatus*) from the Gulf of Mexico. Vet Pathol.

[10] Diaz-Delgado J, Arbelo M, Sacchini S, Quesada-Canales O, Andrada M, Rivero M, Fernandez A (2007). Pulmonary angiomatosis and hemangioma in common dolphins (*Delphinus delphis*) stranded in Canary Islands. J Vet Med Sci.

[11] Lair S, Martineau D, Measures LN. Causes of mortality in St. Lawrence Estuary beluga (*Delphinapterus leuca*) from 1983 to 2012. DFO Can Sci Advis Sec Res Doc. 2014. http://www.dfo-mpo.gc.ca/csas-sccs/publications/resdocs-docrech/2013/2013_119-eng.pdf.

[12] Mawdesley-Thomas LE, Russel FS, Yonge B (1971). Some aspects of neoplasia in marine mammals. Advances in marine biology.

[13] De Guise S, Lagacé A, Béland P (1994). Tumors in St. Lawrence beluga whales (*Delphinapterus leucas*). Vet Pathol.

[14] Fauquier DA, Kinsel MJ, Dailey MD, Sutton GE, Stolen MK, Wells RS, Gulland FM (2009). Prevalence and pathology of lungworm infection in bottlenose dolphins *Tursiops truncatus* from southwest Florida. Dis Aquat Organ.

[15] Sureban SM, May R, Lightfoot SA S, Hoskins AB, Lerner M, Brackett DJ, Postier RG, Ramanujam R, Mohammed A, Rao CV, Wyche JH, Anant S, Houchen CW (2011). DCAMKL-1 regulates epithelial-mesenchymal transition in human pancreatic cells through a miR-200a-dependent mechanism. Cancer Res.

[16] Li M, Luan F, Zhao Y, Hao H, Yu Z, Han W, Fu X (2015). Epithelial-mesenchymal transition: an emerging target in tissue fibrosis. Exp Biol Med.

[17] Fonseca-Alves CE, Kobayashi PE, Rivera-Calderon LG, Laufer-Amorim R (2015). Evidence of epithelial-mesenchymal transition in canine prostate cancer metastasis. Res Vet Sci.

[18] Folberg R, Maniotis AJ (2004). Vasculogenic mimicry. APMIS.

[19] Li Y, Sun B, Zhao X, Zhang D, Wang X, Zhu D, Yang Z, Qiu Z, Ban X (2015). Subpopulations of uPAR+ contribute to vasculogenic mimicry and metastasis in large cell lung cancer. Exp Mol Pathol.

[20] Clemente M, Perez-Alenza MD, Illera JC, Pena L (2010). Histological, immunohistological, and ultrastructural description of vasculogenic mimicry in canine mammary cancer. Vet Pathol.

